# Crystallization of Polylactic Acid with Organic Nucleating Agents under Quiescent Conditions

**DOI:** 10.3390/polym16030320

**Published:** 2024-01-24

**Authors:** Peng Gao, Saeed Alanazi, Davide Masato

**Affiliations:** 1 Department of Plastics Engineering, University of Massachusetts Lowell, Lowell, MA 01854, USA; saeed_alanazi@student.uml.edu; 2 Department of Engineering and Design, Western Washington University, Bellingham, WA 98225, USA

**Keywords:** polylactic acid (PLA), organic nucleating agent, crystallization behavior, quiescent conditions, differential scanning calorimetry

## Abstract

Polylactic acid (PLA) is a versatile and sustainable polymer used in various applications. This research explores the use of orotic acid (OA) and ethylene bis-stearamide (EBS) as nucleating agents to enhance the quiescent crystallization of PLA within the temperature range of 80 °C to 140 °C. Different blends were produced via melt processing before analyzing via DSC, XRD, and SEM. Our results show that both nucleating agents significantly accelerated the crystallization process and reduced the incubation time and the crystallization half-time. The most promising results were obtained with 1% EBS at 110 °C, achieving the fastest crystallization. The XRD analysis showed that at 80 °C, the disordered α’phase predominated, while more stable α phases formed at 110 °C and 140 °C. Combining the 1% nucleating agent and 110 °C promotes densely packed crystalline lamellae. The nucleated PLA exhibited a well-organized spherulitic morphology in agreement with the Avrami modeling of DSC data. Higher nucleating agent concentrations yielded smaller, more evenly distributed crystalline domains. Utilizing OA or EBS in PLA processing could offer enhanced properties, improved processability, and cost-efficiency, making PLA more competitive in various applications.

## 1. Introduction

Biobased polymer materials have gained increased attention during the last decade with various applications, including medical devices, rigid packaging, and agriculture. Biobased materials are derived from renewable resources such as plants, agricultural residues, and algae. Unlike fossil-based materials, the production and decomposition of biobased materials generally result in lower net greenhouse gas emissions [1,2,3]. Biobased materials often have lower levels of toxic additives and chemicals than their conventional counterparts. This reduces the pollution and health risks associated with manufacturing, use, and disposal. Additionally, some biobased materials can be part of circular economies, where products are designed for reuse, recycling, or composting. This minimizes waste and extends the lifespan of materials, contributing to a more sustainable consumption pattern. Among these materials, polylactic acid (PLA) is the most promising.

PLA is a biobased and biodegradable material from renewable resources such as starch and wheat. PLA refers to a family of materials that share slightly different characteristics. PLA has two stereoisomers, namely the L-lactic and the D-lactic acid. The three forms of PLA that are commercially available include the following: pure L-lactide, pure D-lactide, and a mix of L and D-lactide (meso-lactides). Relatively pure L-feed and D-feed PLA are referred to as PLLA and PDLA, respectively [4]. Typical commercial grade PLA with high crystallinity contains a majority of L-feed mixed with a minimum of 1–2% D-feed content, whereas the amorphous grades may contain up to 20% D-feed.

A significant drawback limiting PLA applications is the slow and uncontrollable crystallization kinetics. Indeed, high and controlled crystallization in the final product can significantly impact the mechanical behavior of this product. Harris et al. showed that PLA with 20% crystallinity could achieve 20% higher flexural modulus than the same amorphous PLA. As the crystallinity further increased to 40%, the flexural modulus increased by another 25% [5].

Four crystalline forms of PLA can be developed in PLA depending on the composition and crystallization conditions. PLA’s most common crystalline structure is α form [6,7], which crystallizes from the melt or solvents under quiescent conditions. The disordered α form, known as the α′ crystal, is formed from a melt or solvent but at a lower temperature (<120 °C) [8,9,10]. The β form is usually formed under high shear forces and temperatures [11,12]. The γ crystal is mainly obtained from the hexamethylbenzene substrate via epitaxial growth and is rarely observed in other conditions [13]. Recently, a unique crystal form, the stereocomplex crystal (SC-crystal), received significant attention. The melting point of SC-crystal is 230 °C, which is 50–70 °C higher than the other PLA crystal forms [13,14]. Recent research by Han et al. and Ma et al. shows that the formation of SC crystals is an effective method to enhance the physical and mechanical properties of PLA products [15,16].

The crystallinity of PLA can be enhanced by employing methods such as isothermal annealing [17,18,19,20,21], polymer blending [4,22,23,24,25,26], and strain-induced crystallization during processing [27,28,29,30,31,32,33,34,35]. Isothermal annealing at temperatures of 85–115 °C for an extended time has been reported to initiate and develop the crystalline domains for injection-molded amorphous PLA samples [21,28].

Nucleating agents have been used to increase the nuclei density and crystallization rate. Mineral-based inorganic nucleating agents have been used to enhance the crystallization behaviors of PLA. It was demonstrated that the nucleation density of PLA can be increased by 600% with the addition of 6% talc [36]. Carbon nanotubes (CNTs) were also identified as efficient nucleating agents to promote the crystallization behaviors of PLA. Through polymer grafting, 5–10 wt.% PLA-g-CNT can increase the degree of crystallinity of PLA by 12–14% and reduce the half-crystallization time (t_1/2_) from 4.2 min to 1.9 min [37,38,39].

Compared to inorganic nucleating agents, the focus on organic nucleating agents has increased more recently. Many organic additives are derived from renewable sources and are more likely to be biodegradable than inorganic ones. Organic additives can also enhance the biocompatibility of PLA products. Qiu et al. observed that the crystallization density of PLLA increased by >200% by adding 0.3 wt.% of orotic acid (OA) [40]. Gao et al. observed that 1 wt.% of OA can reduce the crystallization time of PLA from 80 min to <5 min and reduce the energy barrier required for crystallization from 90 °C to 70 °C during the injection molding process [41].

EBS (ethylene bis-stearamide) is a synthetic wax-like compound commonly used as a processing aid and lubricant in various industries. It is derived from the reaction of ethylenediamine with two molecules of stearic acid, resulting in a long-chain amide compound. However, EBS has not been widely recognized as nucleating agents for PLA and other biopolymers [42,43]. Harris et al. reported that 2% of EBS increased the crystallization of PLA3001D from NatureWorks to 18% compared to 10% for neat PLA. In total, 2% of EBS was also reported to decrease the annealing time required by 50% for PLA3001D to reach 40% crystallinity [5]. Limited research has focused on the effect of different concentrations of organic nucleating agents. Moreover, studies used different polymer processing techniques, which can affect crystallization through shear and are, thus, not directly comparable.

In this research, the efficiency of two organic nucleating agents, orotic acid (OA) and ethylene bis-stearamide (EBS), are investigated using differential scanning calorimetry (DSC) isotherm studies at different isotherm temperatures. The objective of this work is the analysis of crystallization dynamics and the morphology obtained under quiescent conditions (i.e., without the introduction of any shear effects). A pure commercial-grade PLA was selected for this research. The degree of crystallinity, crystallization rate, and incubation time are investigated on neat PLA, the PLA-OA blend, and the PLA-EBS blend with different concentration levels. The phases and morphology of the crystalline structures obtained in the PLA samples were examined using X-ray diffraction and directly observed via scanning electron microscope (SEM).

## 2. Materials and Methods

### 2.1. Material Selection

A PLA crystallization investigation was conducted using Ingeo 2500HP obtained from NatureWorks LLC (Plymouth, MN, USA). The extrusion grade of PLA contains PLLA blended with <2% PDLA and can be fabricated into semi-crystalline samples. The material, provided in pellet form, was dried at 40 °C for 12h before any characterization. Table 1 summarizes the main properties of the commercial PLA.

Two nucleating agents were considered to promote crystallization as follows: (1) 97% anhydrous orotic acid (OA) from MilliporeSigma (Burlington, MA, USA), and (2) *N,N’*-ethylene bis stearamide (EBS) from Acme-Hardesty Co. (Blue Bell, PA, USA). Both nucleating agents were received as white powders and dried separately at 100 °C for 2h. The nucleating agents were mixed with the PLA according to the concentrations shown in Table 2. The batch names indicate the selected nucleating agent and the weight concentration.

### 2.2. Sample Preparation

The neat PLA was compounded with additives and then quiescently crystallized into the samples. These samples were directly characterized by their crystallization kinetics and morphology. The cryo-fracturing technique and chemical etching were used to create a fractured surface for the direct observation of crystalline structures. Details about each step of the sample preparation procedure are introduced below.

#### 2.2.1. Compounding

The PLA and nucleating agents were mixed using a static batch mixer (C.W. Brabender Intelli-Torque Plasti-corder, C.W. Brabender Inc., South Hackensack, NJ, USA). For each batch (cf. Table 3), 50 g of neat PLA and a specific amount of the nucleating agent were dry-mixed and then fed into the mixing chamber. A flat temperature profile of 200 °C was used. Each sample was processed at a rotational speed of 100 rpm for 5 min after stabilizing the mixing torque to ensure uniform mixing. The mixed samples were collected and stored in sealed bags at the end of the run. In between runs, a purging compound (Dyna-purge D2, Dyna-purge, Buffalo, NY, USA) was used to clean the mixing chamber.

#### 2.2.2. Quiescent Crystallization

The compounded samples were shaped into disks under quiescent isothermal conditions to allow morphological characterization. The samples were cut into smaller pieces (~10 g) and then arranged in aluminum pans (diameter 70 mm, height 10 mm) to avoid any shear effect on the material. The samples were placed between two hot plates (4394, Carver Compression Molder, Carver, Inc., Wabash, IN, USA), and the temperature was initially stabilized at 220 °C. No pressure was applied. After 60 min, water cooling was used to cool the samples to an isotherm temperature value, which kept below the crystallization point. Three different isotherm temperatures (i.e., 80 °C, 110 °C, and 140 °C) were investigated. The isotherm time was selected to be 60 min to ensure sufficient crystallization. Overall, a total of 21 disk-shaped samples were fabricated and further prepared for characterization.

#### 2.2.3. Sample Etching Procedure

The samples were cryo-fractured to create a flat cross-sectional area to allow the observation of crystal morphology. Each sample was first notched (depth: 1–2 mm) using a bench saw, then kept in a freezer at −20 °C for more than two hours before cracking using pliers. The fractured samples were then chemically etched to expose the crystal structures according to the following procedure:

- In total, 5 g of sodium hydroxide (NaOH), purchased from Sigma-Aldrich (Millipore Sigma, Saint Louis, MO, USA), was dissolved in 250 mL of water to achieve a 0.5 mol/L concentration. Twenty-one clean glass bottles with lids were prepared to etch and hold the PLA samples. The liquid prepared was distributed into 21 bottles.
- Etching: the 21 PLA samples prepared with quiescent crystallization were immersed individually into the solution in glass bottles for 12 h.
- Cleaning: After etching, samples were kept in the bottles for 20 min at 25 °C in an ultrasonic bath (Branson CPX 2800H, Brookfield, CT, USA) to remove residual particles. After cleaning, the samples were removed from the etching solvent and dried with compressed air. The samples were then kept in sealed bags individually for further characterization.

### 2.3. Characterization Techniques

#### 2.3.1. Isothermal Differential Scanning Calorimetry

**Figure 1:**
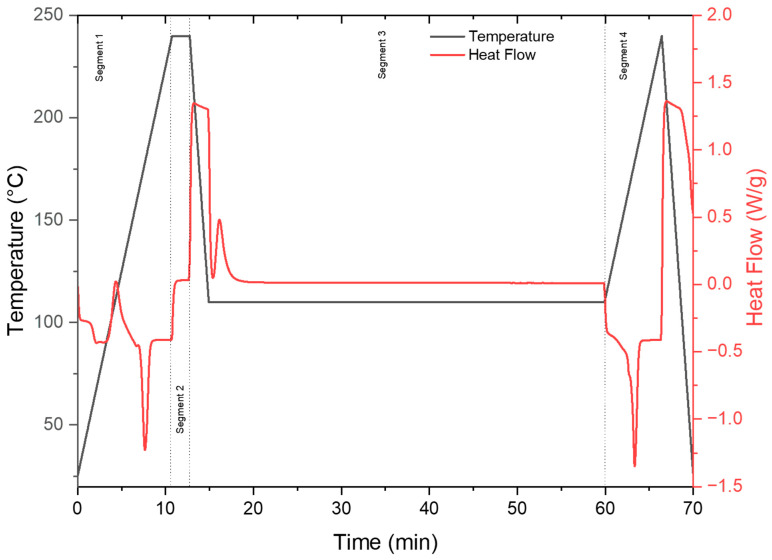
DSC procedure for the characterization of isotherm crystallization.

Differential scanning calorimetry (DSC, 3+ system, Mettler Toledo, Columbus, OH, USA) was used to gain a fundamental understanding of the phase transitions and the melting behavior of the different samples. Fresh samples weighing 5.0–8.0 mg were prepared from the edges of the disk-shaped samples for each DSC run at all isotherm temperatures. Of particular interest was the effect of different concentrations of nucleating agents on the crystallization of PLA. A DSC isothermal test protocol (cf. Figure 1) was defined according to the following steps:

- Segment 1: Heat the sample from 25 °C to 240 °C at 20 °C/min, followed by an isotherm at 240 °C for 3 min. This segment was intended to melt and remove all thermal history of the pellets. The 3 min isotherm ensured the complete melting of the sample.
- Segment 2: Cool the sample rapidly using the maximum cooling rate at 60 °C/min to various isothermal temperatures (80 °C, 110 °C, and 140 °C). This segment is intended to quench the PLA polymer melt to the designed isotherm temperature using the maximum cooling rate, thus minimizing the crystallization behavior during the cooling period.
- Segment 3: The abovementioned isotherm temperatures are held for 60 min. This segment was intended to capture the crystallization process of PLA even with low concentrations of additives and at low temperatures. The 60 min holding time ensures no additional crystallization at the current temperatures and concentrations.
- Segment 4: Heat the sample at 10 °C/min to 240 °C. The degree of crystallinity achieved from the isotherm was quantified from the melting peak observed during this heating segment. The degree of crystallinity (*X_C_*) was calculated using the following:

(1) $$X_{C}=\frac{{∆H}_{m}-{∆H}_{c}}{{∆H}_{M}}\times 100$$

where ${∆H}_{m}$ is the melting enthalpy [J/g], ${∆H}_{c}$ is the cold crystallization enthalpy [J/g], and ${∆H}_{M}$ is the melting enthalpy of a PLA crystal of infinite size. The latter was assumed to be 93 J/g, as obtained from the literature [48].

All DSC experiments were conducted on the PLA-OA, and PLA-EBS compounded using the static batch mixer. Different samples obtained from each batch were tested using preliminary DSC tests with only one heating cycle from 20 to 240 °C, and each batch presented similar melting points and melting peaks, hence suggesting homogeneous mixing. The isotherm DSC experiments were conducted for each batch without replication.

The kinetics of the crystallization process, observed from the DSC experiments, were quantified using the following Avrami equation:

(2) $$v_{c}=1-\exp\left[-K{\left(t\right)}^{n}\right]$$

where $v_{c}$ is the volumetric fraction of the converted phase at time *t*; *K* is the crystallization rate constant; and *n* is the Avrami index. However, the Avrami equation only describes the crystallization process after it has been initiated and does not account for the incubation time. The incubation time highly depends on the temperature and the polymer material [49,50,51]. In this work, the effect of the incubation time on PLA crystallization was quantified using the time derivative of the modified Avrami equation [41]:

(3) $$\frac{dQ}{dt}=∆H_{c}\cdot K\cdot \exp\left[-K{\left(t-t_{0}\right)}^{n}\right]\cdot n\cdot {\left(t-t_{0}\right)}^{n-1}$$

where $\frac{dQ}{dt}$ is the heat flow obtained from the DSC isotherm test, $∆H_{c}$ is the enthalpy of crystallization per unit mass, *K* is the crystallization rate constant, $t_{0}$ is the incubation time, and *n* is the Avrami index for isotherm crystallization.

#### 2.3.2. X-ray Diffractometry

The crystalline phase morphology and the distance between adjacent crystal planes were characterized via X-ray diffractometry (XRD, Rigaku SmartLab II, Rigaku USA, The Woodlands, TX, USA). The XRD experiments were performed using Bragg–Brentano geometry (Cu-Kα source, 1.54184 Å, 40 kV, 50 mA). A step scan protocol with a step size of 2*θ* = 0.002° and a scanning speed of 2*θ* = 5.0°/min was defined. An area of the 5 mm × 5 mm section at the center of each sample was scanned. The Cu-Kα2 signal was removed from the raw signal numerically and corrected for any shift in the diffraction angle. A Pearson VII peak function was utilized to fit the peaks and extract quantitative peak data, such as the peak center, full width at half maximum (FWHM), etc. [52].

(4) $$f\left(x\right)=a{\left[1+\frac{{\left(x-d\right)}^{2}}{b^{2}}\right]}^{-m}$$

where *a* is the maximum height of the peak, *d* is the peak’s center, *b* is proportional to the full width at half-maximum (FWHM), and *m* is the shape factor. When the exponent *m* = 1, the shape becomes Cauchy; *m* = 2, modified Lorentzian; *m* = ∞, Gaussian [53].

The distance between adjacent crystal planes was calculated using Bragg’s Law:

(5) $$n\lambda =2d\;sin\left(\theta \right)$$

where *n* is an integer representing the order of the diffraction peak, *λ* is the wavelength of the incident radiation, *d* is the lattice distance spacing between adjacent crystal planes, also known as d-spacing, and *θ* is the angle between the incident X-ray beam and the crystal plane.

#### 2.3.3. Scanning Electron Microscopy

The morphology of the PLA samples was characterized using scanning electron microscopy (SEM, JEOL JSM 6390, JEOL USA, Inc., Peabody, MA, USA). Before imaging, the etched samples were sputter-coated (Denton Vacuum Desk IV Sputter Coater, Denton North America, Moorestown, NJ, USA) using gold with a 3–4 nanometer thickness. An acceleration voltage of 10 kV and a working distance of 15 mm was selected for all SEM experiments. For each sample, multiple spots were scanned to ensure consistency. For each spot, micrographs were taken at 750×, 1500×, and 3000× magnifications to capture different levels of detail on the crystalline domains. The micrographs were used to observe crystal structures on fractured surfaces. The crystalline domain sizes were measured on the 750× and 1500× micrographs using the image processing software ImageJ (National Institute of Health, Bethesda, MD, USA).

## 3. Results and Discussion

### 3.1. Thermodynamics of Crystallization

#### Degree of Crystallinity

**Figure 2:**
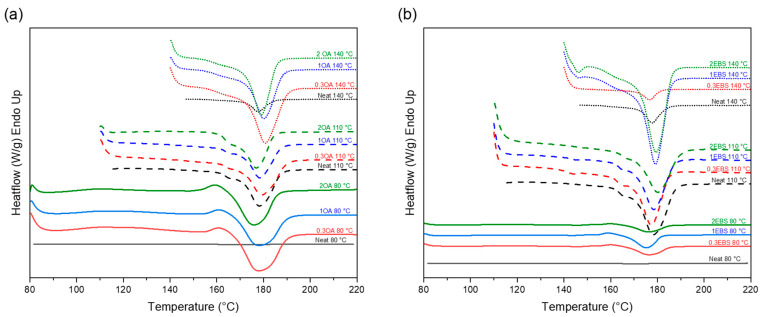
DSC curve for PLA-OA and PLA-EBS samples; the curves show the second heating process (**a**) neat PLA and PLA-OA blends, and (**b**) neat PLA and PLA-EBS blends. Detailed plots for PLA-OA and PLA-EBS can be found in Figures S1 and S2 in the Supplementary Materials.

The results for the degree of crystallization, calculated from Segment 4 in the isotherm DSC, for the different samples are summarized in Table 3. The second heating behaviors for PLA-OA and PLA-EBS blends are presented in Figure 2.

At 80 °C, the isotherm temperature is below the optimal crystallization temperature for the selected PLA grade (i.e., 103–140 °C) [41]. However, it can be observed that with the aid of OA, the degree of crystallization increased from 7.9% to 35.5% as the concentration of the nucleating agent increased from 0.3% to 2%. This indicates that the degree of crystallinity of PLA is highly affected by the concentration of OA, even at the isotherm temperature below the optimal range. For PLA-EBS blends, the nucleating agent concentration seemed to have a smaller effect on the degree of crystallinity at 80 °C (i.e., maintained at 6.9–8.8%).

When the isotherm temperature increased to 110 °C, the degree of crystallinity increased significantly for both OA and EBS blends compared to the degree of crystallinity achieved at 80°C. A further increase to 140 °C resulted in a higher degree of crystallinity. However, an exception was observed for the PLA-0.3EBS at 140 °C, for which the degree of crystallinity decreased significantly to 4.6%.

**Figure 3:**
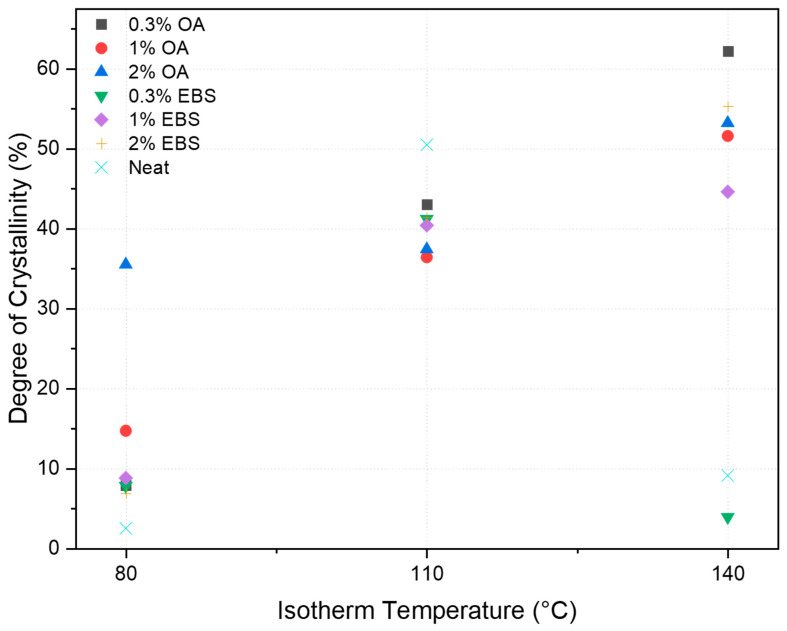
Effect of isotherm temperature and concentration on degree of crystallinity.

Figure 3 presents the effect of the isotherm temperature and concentration of the nucleating agent on the degree of crystallinity. For OA blends, the degree of crystallinity followed an increasing trend as the isotherm temperature increased from 80 °C to 140 °C, and the concentration affected the 80 °C samples significantly. The degree of crystallinity increased as the concentration increased. However, for EBS blends, a low nucleating agent concentration negatively affected the samples prepared at higher isotherm temperatures.

The neat PLA offered the highest degree of crystallinity (i.e., 50.5%) at 110 °C. At the same temperature, EBS and OA blends could not reach the same degree of crystallinity. In fact, nucleating agents enhanced the polymer’s crystallization kinetics and promoted the formation of smaller and more numerous crystalline structures, which could result in more amorphous appearances due to a lack of growth of crystalline domains [21]. However, at lower temperatures (i.e., 80 °C), the degree of crystallinity increased from 6.5% for neat PLA to a maximum of 35.5% for PLA-2OA. Overall, the nucleating agents expanded the processing window for the PLA, which has relevant implications for manufacturing. Indeed, the ability to increase the crystallization at lower temperatures significantly facilitates processing by allowing the use of a water-heating system. Moreover, the energy consumption used for heating is reduced.

When comparing batches, the melt temperature of the PLA-EBS samples showed a ~4 °C decrease compared to the PLA-OA ones at the same isothermal temperatures. The change in melt temperature was minimal but might affect the crystalline morphology and adhesion between crystalline structures. It was also observed that at 80 °C, all DSC curves showed a unique endothermic peak at ~160 °C. For all PLA-OA blends, the intensity of this exothermic peak remained similar. However, for the PLA-EBS samples, the intensity decreased as the concentration increased from 0.3% to 2%. The same endothermic behavior was observed by other researchers [22,54,55] and could be related to the melting of α’ PLA crystals. In general, α’ crystals are a less stable form of crystalline PLA. They have a less ordered and less dense molecular arrangement than α crystals. α’ crystals have a lower melting temperature and mechanical strength than the α form. These crystals are formed when PLA chains have less packing density and exhibit some chain dislocations or imperfections. The non-ideal crystallization temperature, low cooling rate, and the absence of nucleating agents promote the formation of α’ crystals. The melting of α’ crystals is also observed in all DSC curves, represented by the endothermic peak in the 142–155 °C range. When the DSC scanning temperature surpasses their melting temperature, the α’ crystals start to dissolve or transform into the more stable α phase, represented by the exothermic peaks observed at ~160 °C. The absence of this unique exothermic peak indicates that there is no α’ crystal existing at 160 °C that can be transformed into α-phase crystals. All α’ crystals formed during the isothermal segment were melted at 142–155 °C. The subsequent XRD experiments further investigated the formation of α’ crystals and α crystals.

The crystallization kinetics were calculated according to the exothermic peaks in Segment 3 (cf. Figure 1). The heat flow values were baseline corrected using a tangential baseline before being treated, and Equation (3) was fitted to obtain the crystallization rate, the Avrami index, and the incubation time. The relative degree of crystallization values was obtained from integrating the heat flow data. The crystallization kinetics for PLA-OA and PLA-EBS blends are summarized in Table 4.

The crystallization peaks for both PLA-OA and PLA-EBS blends indicated that the crystallization behavior is more intense at 110 °C than at 80 °C and 140 °C. For PLA-OA blends, the initial crystallization rate was found to be the lowest at 80 °C for PLA-0.3OA, and the highest crystallization rate was 1.482 min^-n^ at 110 °C for PLA-2OA. The crystallization rate increased by two or more orders of magnitude at 110 °C. At 110 °C, there were no significant thermal behaviors after ~500 s during the isotherm segment. However, the crystallization behavior was not completed after 40 min at 80 °C. The initial crystallization rate also increased with a higher OA concentration.

The crystallization half-time (t_1/2_), the time a sample takes to reach 50% relative crystallinity, provides critical crystallization information for process optimization and predictive modeling. Indeed, it captures the effect of both initial nucleation and the growth of crystalline domains, which occur in the later stage of crystallization behavior. It also provides more general information about the crystallization process and is easier to obtain than the crystallization rate (*k*) (cf. Equation (3)). The crystallization half-time indicated that PLA-2OA and the isotherm temperature of 110 °C provided the fastest nucleation and growth. However, the differences between different concentrations were minimal. The crystallization half-time changed from 1.4–1.61 min at 110 °C to 3.7–4.6 min at 140 °C.

The isotherm DSC results showed that EBS blends were characterized by a higher degree of crystallinity, a faster initial crystallization rate, lower incubation time, and shorter crystallization half-time. The PLA-EBS blends also showed sharper and narrower crystallization peaks at 110 °C compared to 80 °C and 140 °C (cf. Figures S3 and S5 in Supplementary Materials). Unlike PLA-OA blends, the initial crystallization rates were the highest at 1% EBS at 80 °C and 110 °C, while at 140 °C, the crystallization rate was the lowest at 1%. However, the PLA-1EBS blend showed a wider crystallization peak, indicating that crystallization growth was enhanced at this condition. The degree of crystallization was 44.6% for PLA-1EBS at 140 °C. This also confirmed that even though the initial crystallization rate was lower, the intensity of molecular movement was significant enough for the nucleation sites to grow into crystalline structures.

**Figure 4:**
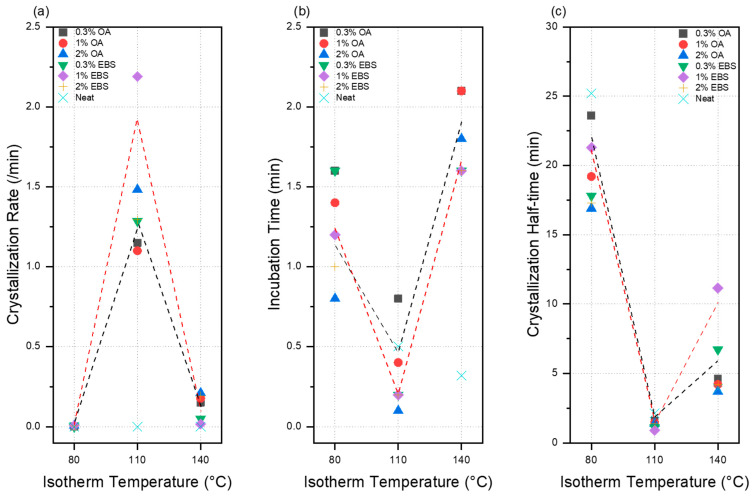
Effects of isotherm temperature and concentration on different crystallization kinetics, (**a**) crystallization rate, (**b**) incubation time, and (**c**) crystallization half-time. The black curve indicates the trend line for PLA-OA samples, and the red curve indicates the trend line for PLA-EBS samples.

The effects of isotherm temperature and the concentration of nucleating agents on the crystallization kinetics are shown in Figure 4. At the 110 °C isotherm temperature, the crystallization rate was the fastest, while the incubation time and crystallization half-time were the lowest.

The Avrami index (*n*) provides information about the nucleation and growth mechanism during crystallization. The value of *n* can vary, and different values suggest different crystallization mechanisms as follows:

*n* = 1: Indicates that the crystallization occurs through one-dimensional growth. This suggests that the growth of crystalline structures is linear (e.g., fibric growth, shish-kebab structures).

*n* = 2: Suggests that crystallization occurs through three-dimensional growth. This indicates that the crystalline structure growth is volumetric (e.g., disc-shaped structures, spherulites).

*n* > 2: Suggests heterogeneous nucleation and/or diffusion-controlled growth mechanisms.

The Avrami index for all tested samples was between 1.7 and 2.4, indicating that major crystalline structures were either disc-shaped crystals or spherulites. This observation is also supported by other research [30,56] and following SEM imaging analysis.

### 3.2. Polymorphic Analysis of Crystalline Domains

**Figure 5:**
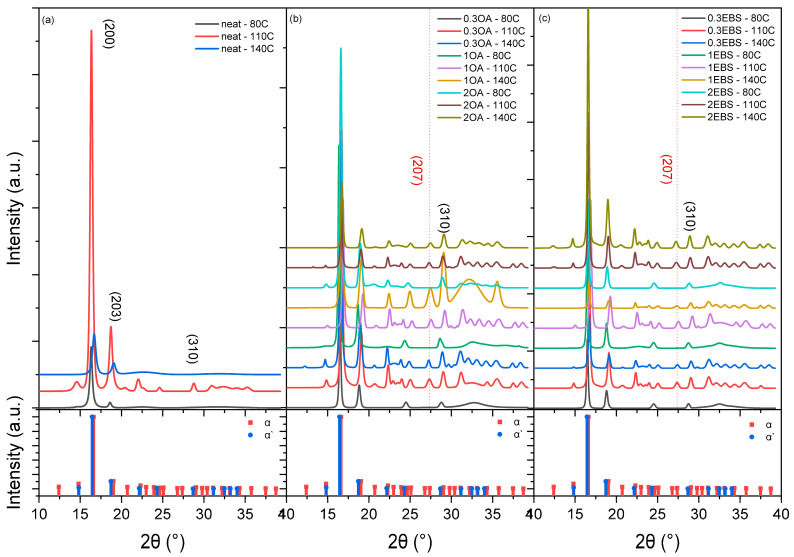
Wide-angle XRD patterns for (**a**) neat PLA, (**b**) PLA-OA, and (**c**) PLA-EBS.

The DSC data in Figure 2 suggest the formation of both α and α’ crystals. The XRD curves of all samples are presented in Figure 5 and can be compared with the XRD patterns for the α and α´phase according to standard files to further prove the phase differences. Neat PLA samples crystallized at the same conditions were characterized as baselines for the raw material. The peak centers of the most significant peaks (200) are summarized in Table 5. The interplanar spacing of the crystal planes was calculated based on the (200) peaks.

**Figure 6:**
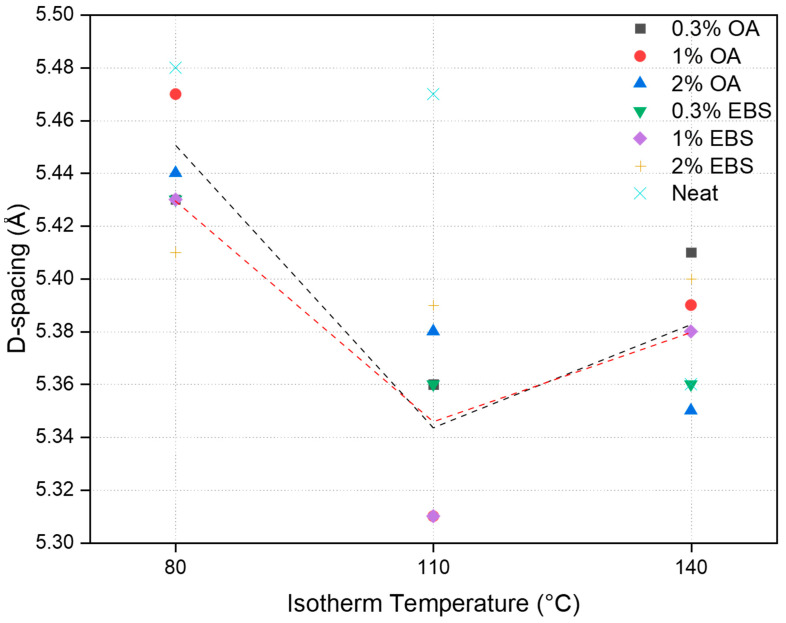
Effects of isotherm temperature and concentration on Basal distance spacing. The black curve indicates the trend line for PLA-OA samples, and the red curve indicates the trend line for PLA-EBS samples.

The peak positions were compared using the JCPDS standards for α (JCPDS#00-064-1624) and α´phases (JCPDS#00-064-1624). The (200) peak for the α phase is centered at 2θ = 16.62° and 2θ = 16.44° for the α´phase. The (200) peak positions of all samples fabricated were between 2θ = 16.34° and 2θ = 16.86°. Despite the systematic errors in the XRD measurements, the crystalline structures in all samples were a combination of the α´phase and α phase. Lower peak positions indicated a more significant amount of α´phase, while higher peak positions suggested more α phase. Additionally, the α phase crystals exhibit unique peaks at the following positions: (204) peak at 2θ = 20.71°, (213) peak at 2θ = 23.92° and (207) peak at 2θ = 27.36°. The peak center positions of the common peaks for α and α´phases are very close and hard to separate numerically. The existence of the α phase was determined by the (207) peak and is indicated by the red dotted lines in Figure 6. The samples showed a significant peak at the (207) peak location, indicating the existence of phase crystals. Otherwise, most crystal structures were α´phase if no peak was observed at the (207) peak position. The XRD scans suggested that the α´phase was the majority phase of the crystals in all neat PLA samples, while PLA-OA and PLA-EBS blends were crystallized at 80 °C. This observation confirms the DSC analysis, where significant exothermic peaks were observed at around 160 °C, representing the α´ to α phase transition during heating.

The d-spacing data, describing the distance between adjacent crystal lattice planes within a crystalline material, of the (200) crystals are presented in Table 5. The standard d-spacing for the (200) crystal in the α´phase is 5.39 Å, and 5.33 Å in the α phase, according to ICDD files. The lowest d-spacing values (5.31 Å) were found on PLA-1OA and PLA-1EBS samples crystallized at 110 °C. The value was smaller than the ICDD standard value, indicating that nucleating agents improved the molecular orientation compared to ICDD conditions, thus resulting in densely packed molecular chains and crystalline structures. Decreasing or increasing the nucleating agent concentration disrupted the well-oriented molecular chains and led to larger d-spacing values.

The isotherm temperature mostly dominated the d-spacing values. Figure 6 presents the effect of the isotherm temperature and concentration on the d-spacing of crystalline domains. This trend showed the smallest d-spacing values at a 110 °C isotherm temperature with 1% of nucleating agents adopted. This indicated that at 110 °C and a 1% nucleating agent concentration, the distance between the PLA crystalline lamellae was the lowest. Hence, the most perfect crystals were formed under this condition.

### 3.3. Crystallization Morphology

The crystallization morphology was characterized using SEM images taken from the same conditions. Table 6 summarizes the SEM images obtained at a magnification of 750×. A micrograph of neat crystallized PLA is shown in Table 7 as a reference.

The radius of individual crystal structures was measured directly from the SEM images at 750× magnification, and the results are summarized in Table 8. All crystalline domains showed a disc/circle shape, indicating that the crystalline structures are spherulites/lamellae in growth. The results confirmed the Avrami index values obtained from the isotherm DSC analysis.

Compared to neat PLA crystals (i.e., radius of 54.5 ± 2.0 μm), all samples fabricated with the nucleating agents had smaller crystalline domain sizes. Additionally, relatively higher nucleation densities were found in all PLA-OA and PLA-EBS samples, indicating that both OA and EBS acted as nucleating agents to provide heterogeneous nucleation sites for PLA spherulitic growth. In all 80 °C samples, the crystalline domain density was lower than 110 °C and 140 °C samples, and only a small amount of crystal structures could be observed. This supported the observation of a low degree of crystalline in the 80 °C samples. In samples crystallized at 110 °C, nucleation density and crystalline domain sizes were relatively high, and the interfaces between crystalline domains were observed on the fractured surfaces. In samples crystallized at 140 °C, the mean crystalline sizes were 6.5 ± 1.0 μm to 11.7 ± 1.4 μm, except for the PLA-0.3EBS sample. The radius of the crystal structure of PLA-0.3EBS was measured as 40.4 ± 5.2 μm, which is closer to the value for neat PLA samples. Additionally, the nucleation density, indicated by the number of individual crystalline domains observed in the SEM images with 750× magnification, was much lower than the PLA-1EBS and PLA-2EBS samples. This also supports the low degree of crystallinity in the PLA-0.3EBS sample. The crystallization condition at 140 °C and low concentration of EBS provided a low crystallization density, and the high crystallization temperature allowed more intense molecular movement, resulting in better molecular alignment. The nucleation density was much higher in PLA-1EBS and PLA-2EBS samples at 140 °C as more nucleation sites were provided. As a result, the sizes of the individual crystalline domains were smaller since there were not enough free volumes for the crystalline domains to grow.

Additionally, the variation in the crystal sizes was significantly lower with PLA-2OA and PLA-2EBS samples compared to PLA-0.3OA and PLA-0.3EBS samples. This also confirmed that the material achieved a state where most of the crystallites reached their thermodynamically favored size at higher concentrations. Under such conditions, the energy barriers for crystal growth and nucleation are balanced, leading to a distribution of relatively similar crystallite sizes. Achieving a state of more stable crystal sizes can improve material properties, such as mechanical strength, thermal stability, and degradation stability. Controlled crystallization processes at higher isotherm temperatures during PLA processing can influence the crystallites’ size and arrangement, impacting the final products’ performances.

Overall, the nucleation domain to initialize the crystallization of neat PLA is (a) meso-lactide and (b) SC-crystals [36,57]. Both nucleation sites are rare in neat PLA due to the high l-lactide in polymerization for the PLA 2500HP and the high-temperature requirement for forming sc-crystals. Orotic acid and EBS serve as nucleating agents for PLA via introducing nucleation sites. As a result, the number density increased, and the sizes of nucleation domains were smaller. Additionally, the hydrogen bonding between the nucleating agent and PLA altered the chemical structures when blended [58]. This also affected the mobility of the molecular chains and the thermodynamics of the crystallization behavior.

## 4. Conclusions

The experiments and analysis reported in this work suggest that both OA and EBS can be effectively used as nucleating agents for PLA. The nucleating agents enhanced PLA quiescent crystallization from 80 °C to 140 °C. OA and EBS significantly increased the initial crystallization rate during quiescent isotherm annealing and reduced the incubation time and crystallization half-time. The fastest crystallization rate and smallest crystallization half-time were achieved in the case of blends containing 1% EBS at 110 °C.

Both nucleating agents efficiently reduced the energy barrier of nucleation by achieving high crystallinity at 80 °C, which is 40 °C lower than the recommended processing temperature for PLA 2500HP. At 80 °C, the disordered α’phase was the primary phase in all samples, while the more stable α phase was obtained in samples annealed at 110 °C and 140 °C. Combining the 1wt.% nucleating agent and isotherm temperature at 110 °C promoted the smallest Basal distance spacing, indicating densely packed crystalline lamellae.

The well-organized and evenly distributed spherulitic morphology of the nucleated PLA crystalline domains was observed on the cryo-fractured surfaces. The SEM micrographs confirmed the calculated Avrami index values from the DSC experiments. It was also observed that increasing the concentration of nucleating agents resulted in the formation of smaller and more evenly distributed crystalline domains.

Overall, the use of OA and EBS led to a higher degree of crystallinity at lower temperatures, smaller and more evenly distributed crystalline structures, a faster crystallization rate, lower crystallization temperature, and lower crystallization half-time when compared to neat PLA. Hence, the blends are expected to improve the processability, reduce energy consumption, and enhance the performance of semi-crystalline PLA products. Future studies should consider the shear-induced effects of manufacturing conditions on the blend morphology.

## Figures and Tables

**Table 1:** Main PLA properties. (* molded crystalline with a 120 °C mold temperature; the formula included 1 wt% of the nucleating agent (LAK-301, Takemoto Oil & Fat, Gamagori-shi Japan)).

Properties	Ingeo 2500HP	ASTM Standard
Specific gravity	1.24	D792 [44]
MFR, g/10 min (210 °C, 2.16 kg)	8	D1238 [45]
Relative viscosity (in 1.0 g/dL chloroform, 30 °C)	4.0	D5225 [46]
Highest crystallization melting point *, °C	160–180	D3418 [47]

**Table 2:** Definition of different batch samples for the experimental investigation.

Batch Name	Nucleating Agent	Concentration (wt.%)
Neat PLA	N/A	N/A
PLA-0.3OA	Orotic acid	0.3
PLA-1OA	Orotic acid	1
PLA-2OA	Orotic acid	2
PLA-0.3EBS	EBS	0.3
PLA-1EBS	EBS	1
PLA-2EBS	EBS	2

**Table 3:** Degree of crystallinity of PLA samples measured from the DSC isotherm.

PLA Batch	Degree of Crystallinity (%)		
	Isotherm Temperature		
	80 °C	110 °C	140 °C
**Neat PLA**	2.5	50.5	9.1
**PLA-0.3OA**	7.9	43.0	62.2
**PLA-1OA**	14.7	36.4	51.6
**PLA-2OA**	35.5	37.4	53.2
**PLA-0.3EBS**	7.8	41.2	3.9
**PLA-1EBS**	8.8	40.4	44.6
**PLA-2EBS**	6.9	41.4	55.3

**Table 4:** Crystallization kinetics of PLA-OA and PLA-EBS blends. Detailed plots can be found in Figures S3–S6 in the Supplementary Materials.

Blends	Isotherm Temperature (°C)	Crystallization Rate (/min^−n^)	Avrami Index	Incubation Time (min)	t_1/2_ (min)
Neat PLA	80	0.0001 ± 2.92 × 10^−6^	1.0 ± 1.2 × 10^−4^	5.86 ± 2.2 × 10^−4^	34.2
Neat PLA	110	0.0012 ± 4.13 × 10^−4^	3.3 ± 0.02	0.5 ± 0.02	2.14
Neat PLA	140	0.002 ± 1.63 × 10^−4^	1.2 ± 0.002	0.32 ± 0.07	3.9
PLA-0.3OA	80	3.6 × 10^−4^ ± 3.7 × 10^−6^	2.0 ± 0.03	1.6 ± 0.2	23.6
PLA-1OA	80	0.0029 ± 3.4 × 10^−5^	1.9 ± 0.004	1.4 ± 0.03	19.2
PLA-2OA	80	0.00814 ± 6.5 × 10^−5^	1.7 ± 0.003	0.8 ± 0.01	16.9
PLA-0.3OA	110	1.149 ± 0.015	1.7 ± 0.02	0.8 ± 0.008	1.61
PLA-1OA	110	1.099 ± 0.029	1.8 ± 0.05	0.4 ± 0.01	1.45
PLA-2OA	110	1.482 ± 0.026	2.2 ± 0.04	0.1 ± 0.01	1.4
PLA-0.3OA	140	0.152 ± 0.006	2.2 ± 0.03	2.1 ± 0.02	4.6
PLA-1OA	140	0.179 ± 0.007	2.0 ± 0.03	2.1 ± 0.02	4.2
PLA-2OA	140	0.211 ± 0.017	2.3 ± 0.06	1.8 ± 0.04	3.7
PLA-0.3EBS	80	8.7 × 10^−4^ ± 1.1 × 10^−5^	2.7 ± 0.3	1.6 ± 0.2	17.8
PLA-1EBS	80	0.0084 ± 7.7 × 10^−4^	1.7 ± 0.01	1.2 ± 0.1	21.3
PLA-2EBS	80	0.0047 ± 0.0024	2.0 ± 0.1	1.0 ± 0.1	17.3
PLA-0.3EBS	110	1.285 ± 0.018	2.4 ± 0.02	0.2 ± 0.01	1.1
PLA-1EBS	110	2.190 ± 0.059	2.1 ± 0.1	0.2 ± 0.02	0.9
PLA-2EBS	110	1.294 ± 0.129	2.4 ± 0.1	0.2 ± 0.05	1.4
PLA-0.3EBS	140	0.050 ± 3.45 × 10^−4^	2.1 ± 0.2	1.6 ± 0.02	6.7
PLA-1EBS	140	0.017 ± 0.004	1.9 ± 0.02	1.6 ± 0.02	11.15
PLA-2EBS	140	0.193 ± 0.001	1.8 ± 0.01	1.6 ± 0.01	4.2

**Table 5:** Peak centers and Basal distance spacing (d-spacing) obtained from XRD measurements of all PLA-OA and PLA-EBS samples according to (200) peaks.

Sample	Isotherm Temperature (°C)	2*θ* (°)	D-Spacing (Å)
Neat PLA	80	16.31	5.48
Neat PLA	110	16.34	5.47
Neat PLA	140	16.67	5.36
PLA-0.3OA	80	16.49	5.43
PLA-0.3OA	110	16.70	5.36
PLA-0.3OA	140	16.55	5.41
PLA-1OA	80	16.34	5.47
PLA-1OA	110	16.85	5.31
PLA-1OA	140	16.61	5.39
PLA-2OA	80	16.45	5.44
PLA-2OA	110	16.63	5.38
PLA-2OA	140	16.72	5.35
PLA-0.3EBS	80	16.47	5.43
PLA-0.3EBS	110	16.69	5.36
PLA-0.3EBS	140	16.70	5.36
PLA-1EBS	80	16.46	5.43
PLA-1EBS	110	16.86	5.31
PLA-1EBS	140	16.64	5.38
PLA-2EBS	80	16.55	5.41
PLA-2EBS	110	16.61	5.39
PLA-2EBS	140	16.57	5.40

**Table 6:** SEM images (750×) of PLA-OA and PLA-EBS samples.

OA	0.3%	1%	2%
80 °C	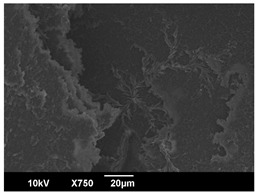	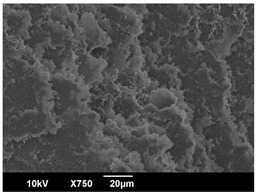	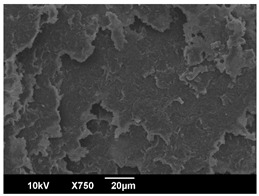
110 °C	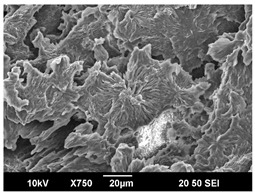	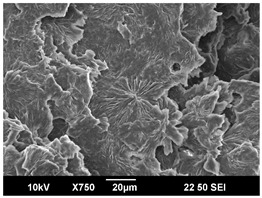	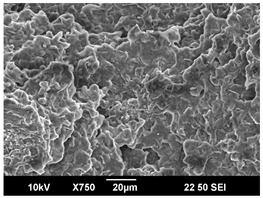
140 °C	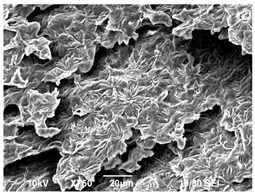	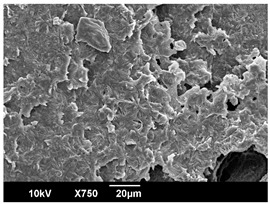	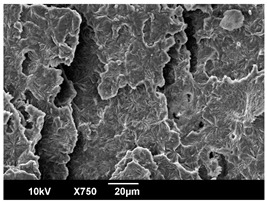
EBS	0.3%	1%	2%
80 °C	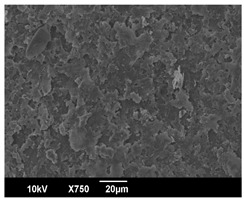	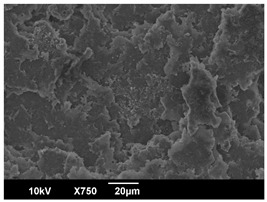	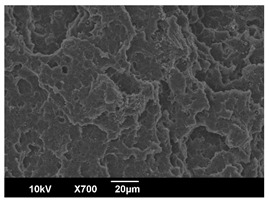
110 °C	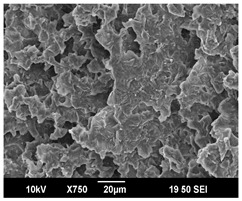	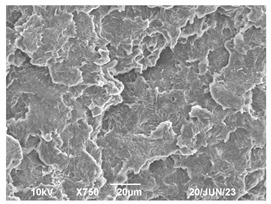	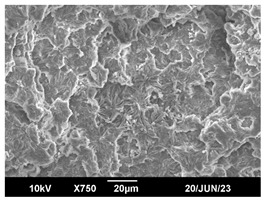
140 °C	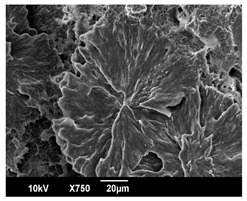	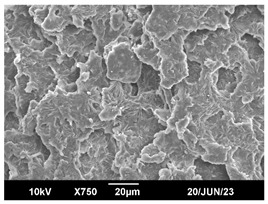	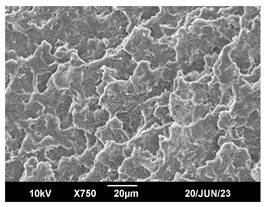

**Table 7:** SEM images (750×) of neat PLA crystallized at 80 °C, 110 °C, and 140 °C for 45 min.

Neat PLA, 80 °C	Neat PLA, 110 °C	Neat PLA, 140 °C
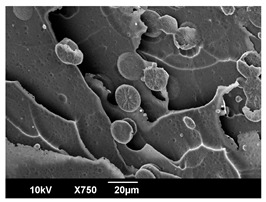	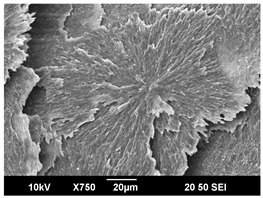	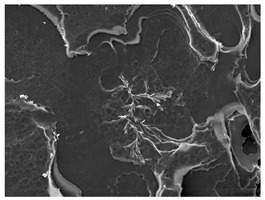

**Table 8:** Radius of crystalline domains in PLA-OA and PLA-EBS samples.

	Isotherm Temperature		
	80 °C	110 °C	140 °C
PLA-0.3OA	7.8 ± 1.0 μm	22.6 ± 5.2 μm	11.7 ± 1.4 μm
PLA-1OA	9.2 ± 0.6 μm	15.6 ± 2.4 μm	7.8 ± 0.7 μm
PLA-2OA	8.8 ± 1.0 μm	10.8 ± 2.0 μm	6.5 ± 1.0 μm
PLA-0.3EBS	7.2 ± 2.4 μm	8.0 ± 2.0 μm	40.0 ± 5.2 μm
PLA-1EBS	9.3 ± 0.5 μm	9.0 ± 1.5 μm	11.5 ± 1.2 μm
PLA-2EBS	10.0 ± 1.5 μm	9.0 ± 1.2 μm	8.0 ± 0.5 μm

## Data Availability

The raw data supporting the conclusions of this article will be made available by the authors on request.

## Supplementary Materials

The following supporting information can be downloaded at: https://www.mdpi.com/article/10.3390/polym16030320/s1, Figure S1: DSC curves for PLA-OA samples. Curves showed melting behavior during the second heating cycle, (**a**) overall, (**b**) 80 °C, (**c**) 110 °C, and (**d**) 140 °C; Figure S2: DSC curves for PLA-EBS samples. Curves showed melting behavior during the second heating cycle, (**a**) overall, (**b**) 80 °C, (**c**) 110 °C, and (**d**) 140 °C; Figure S3: DSC curves for PLA-OA samples. Curves showed heat flow data obtained during isotherm cycle, (**a**) 80 °C, (**b**) 110 °C, and (**c**) 140 °C; Figure S4: Curves for relative degree of crystallinity for PLA-OA samples, (**a**) overall, (**b**) 80 °C, (**c**) 110 °C, and (**d**) 140 °C; Figure S5: DSC curves for PLA-EBS samples. Curves showed heat flow data obtained during isotherm cycle, (**a**) 80 °C, (**b**) 110 °C, and (**c**) 140 °C; Figure S6: Curves for relative degree of crystallinity for PLA-EBS samples, (**a**) overall, (**b**) 80 °C, (**c**) 110 °C, and (**d**) 140 °C.
