# Supplementary material for: Crystallization of Polylactic Acid with Organic Nucleating Agents under Quiescent Conditions
Source: Polymers (Basel). 2024 Jan 24;16(3):320. doi: 10.3390/polym16030320 (PMC10857276; doi:10.3390/polym16030320)
Supplement: Supplementary file 1 [file polymers-16-00320-s001.zip › polymers-2748927-supplementary.pdf]

Supplementary Materials

Crystallization of Polylactic Acid with Organic Nucleating Agents under Quiescent Conditions

Peng Gao [1,2,*], Saeed Alanazi [1] and Davide Masato [1,*]

1 Department of Plastics Engineering, University of Massachusetts Lowell, Lowell, MA 01854, USA; saeed_alanazi@student.uml.edu (S.A.); davide_masato@uml.edu (D.M.)
2 Department of Engineering and Design, Western Washington University, 516 High Street, Bellingham, WA, USA
* Correspondence: gaop@wwu.edu (P.G.); davide_masato@uml.edu (D.M.)

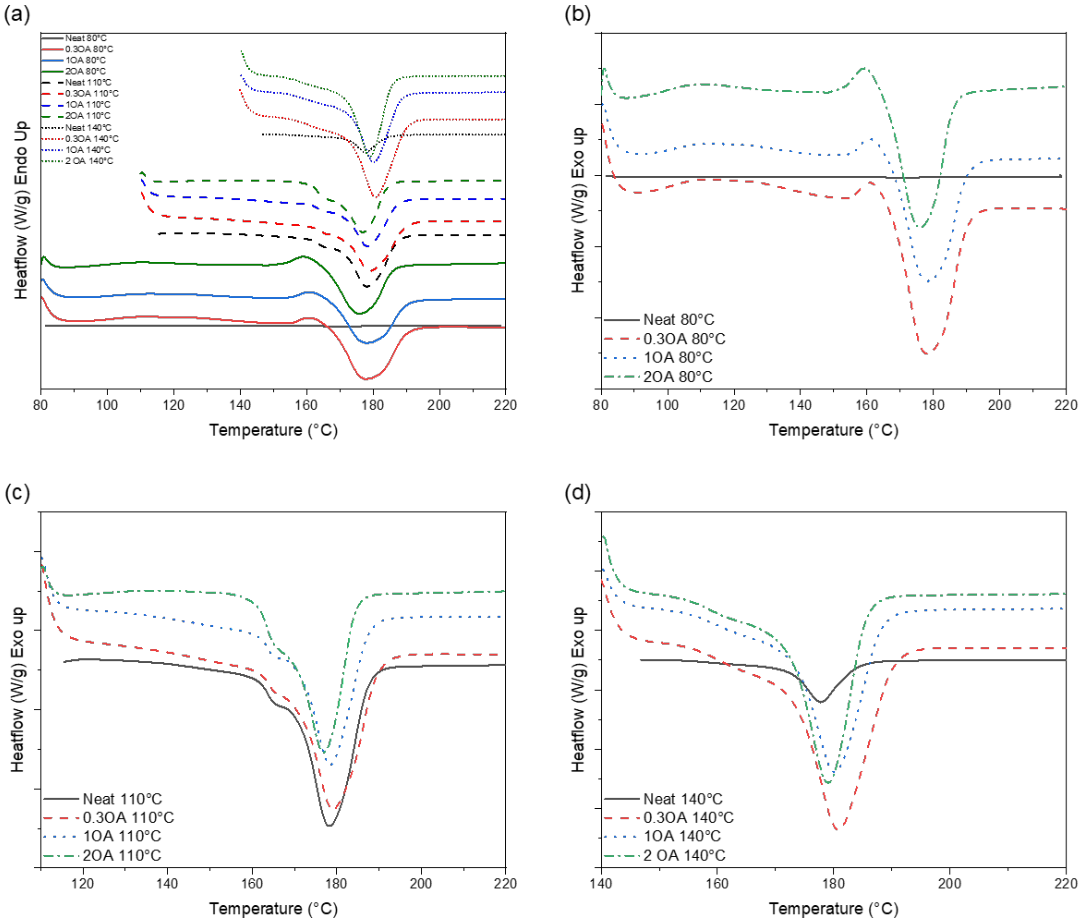

Figure S1: DSC curves for PLA-OA samples. Curves showed melting behavior during the second heating cycle, (a) overall, (b) 80 °C, (c) 110 °C, and (d) 140 °C.

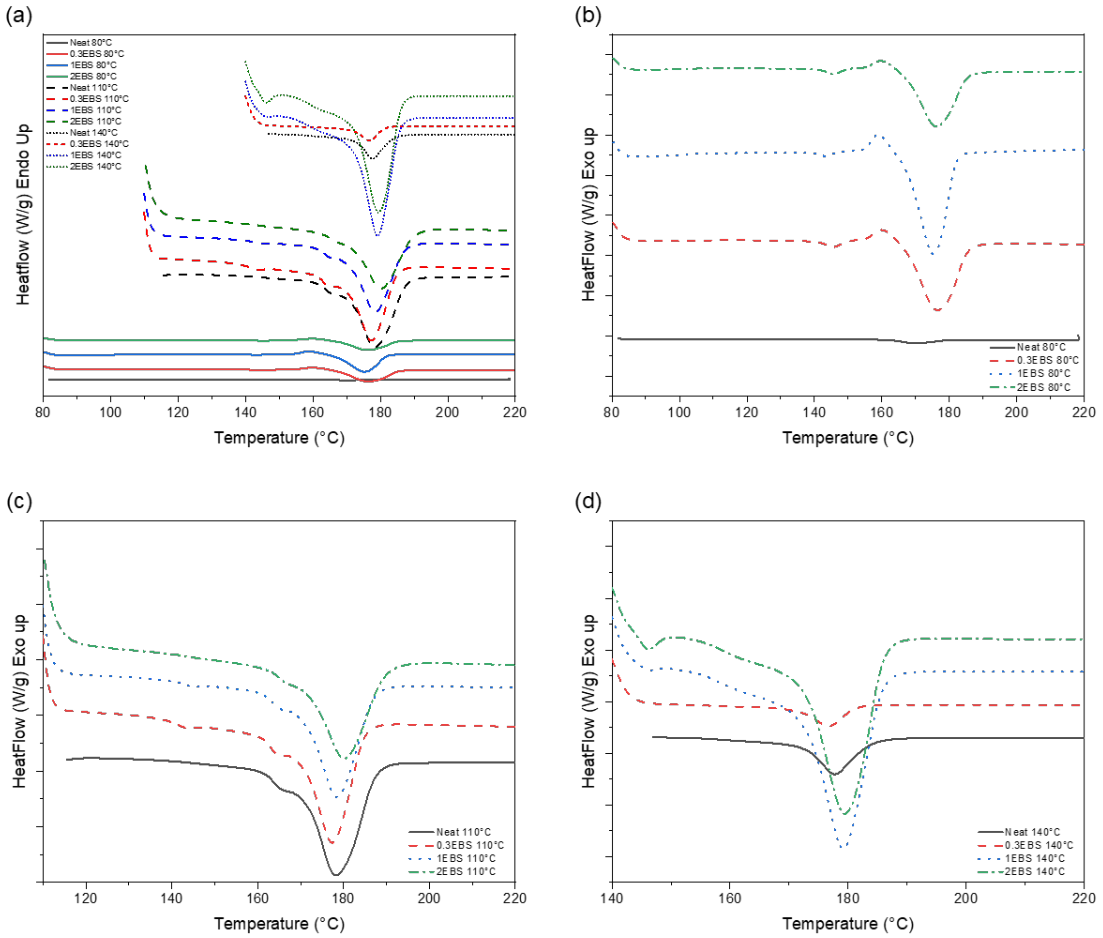

Figure S2: DSC curves for PLA-EBS samples. Curves showed melting behavior during the second heating cycle, (a) overall, (b) 80 °C, (c) 110 °C, and (d) 140 °C.

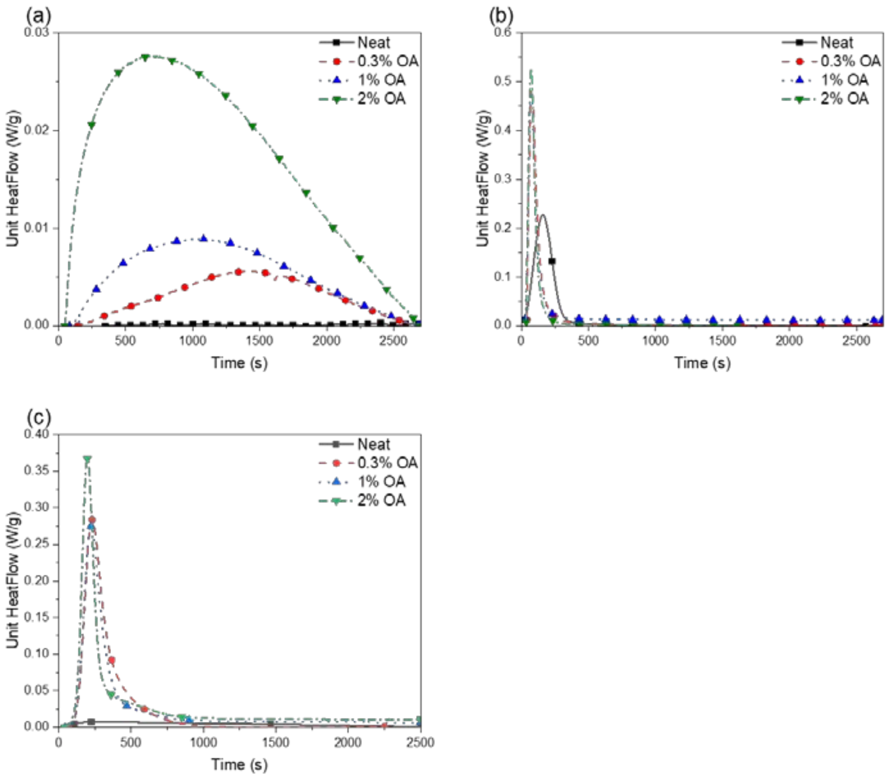

Figure S3: DSC curves for PLA-OA samples. Curves showed heat flow data obtained during isotherm cycle, (a) 80 °C, (b) 110 °C, and (c) 140 °C.

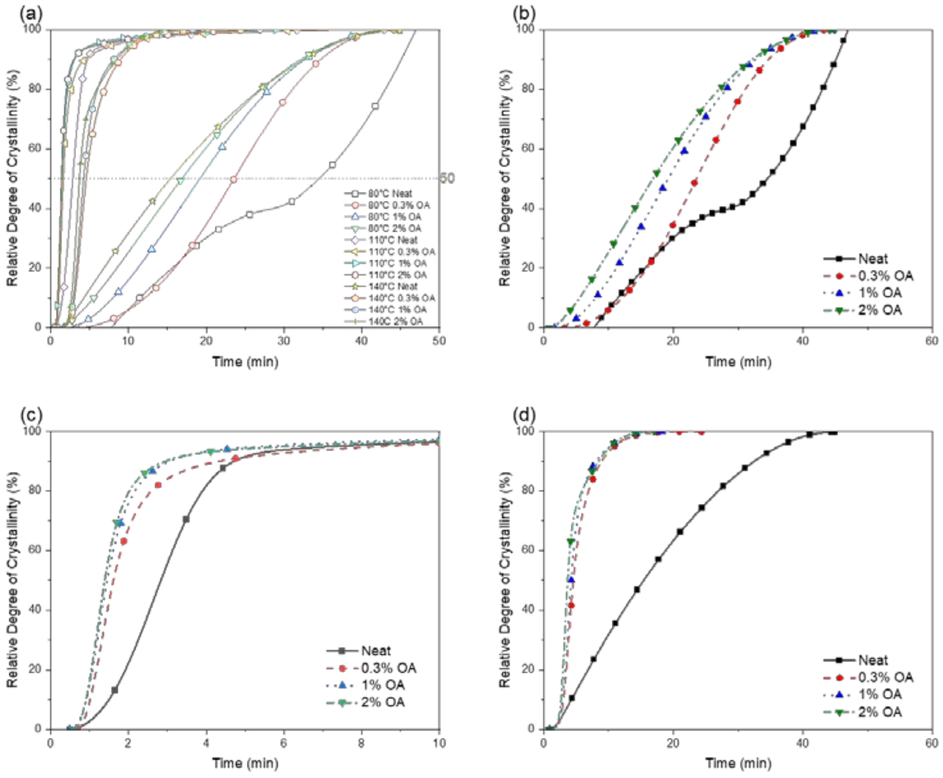

Figure S4: Curves for relative degree of crystallinity for PLA-OA samples, (a) overall, (b) 80 °C, (c) 110 °C, and (d) 140 °C.

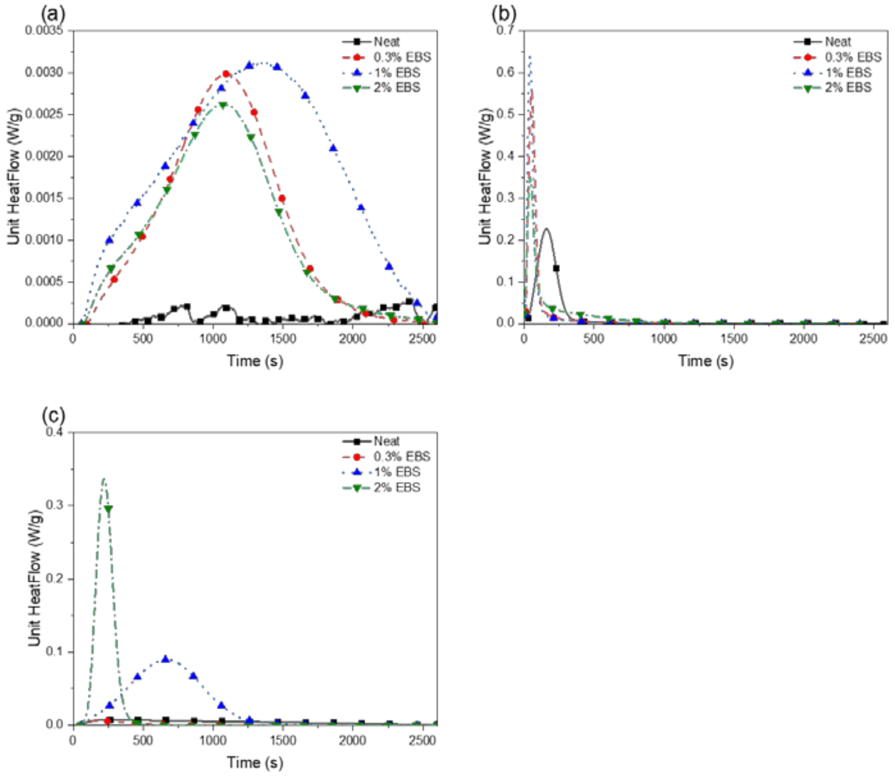

Figure S5: DSC curves for PLA-EBS samples. Curves showed heat flow data obtained during isotherm cycle, (a) 80 °C, (b) 110 °C, and (c) 140 °C.

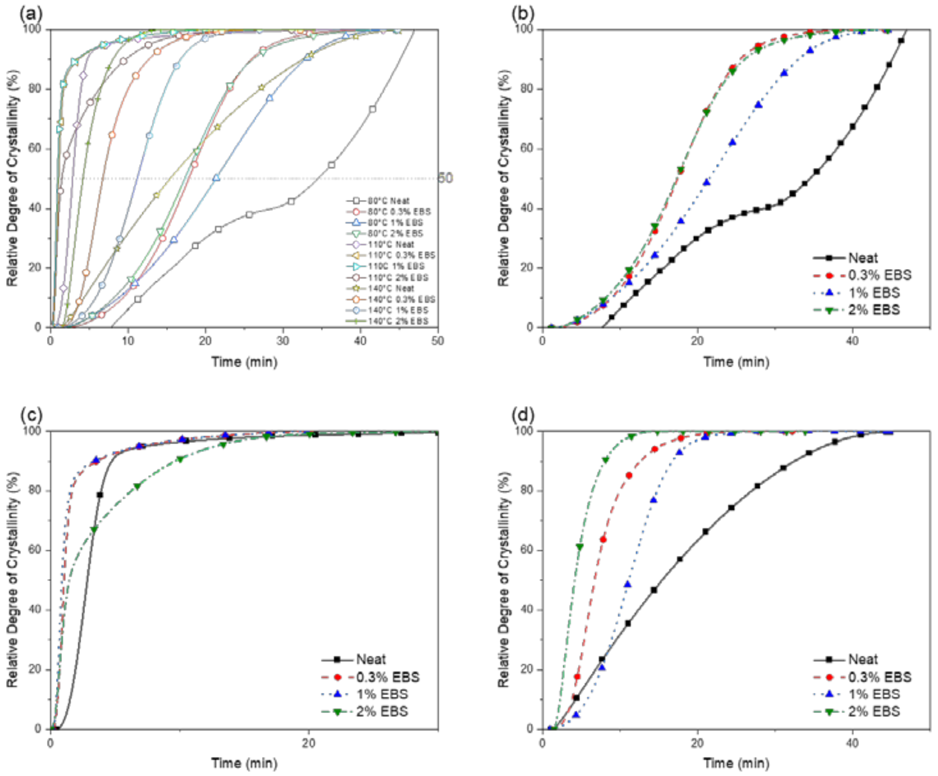

Figure S6: Curves for relative degree of crystallinity for PLA-EBS samples, (a) overall, (b) 80 °C, (c) 110 °C, and (d) 140 °C.
